# Skeletal abnormalities caused by a Connexin43_R239Q_ mutation in a mouse model for autosomal recessive craniometaphyseal dysplasia

**DOI:** 10.21203/rs.3.rs-3906170/v1

**Published:** 2024-02-06

**Authors:** Yasuyuki Fujii, Iichiro Okabe, Ayano Hatori, Shyam Kishor Sah, Jitendra Kanaujiya, Melanie Fisher, Rachael Norris, Mark Terasaki, Ernst J. Reichenberger, I-Ping Chen

**Affiliations:** 1 Department of Endodontology, School of Dental Medicine, University of Connecticut Health, Farmington, CT, United States; 2 Department of Cell Biology, University of Connecticut Health, Farmington, CT, United States; 3 Center for Regenerative Medicine and Skeletal Development, School of Dental Medicine, University of Connecticut Health, Farmington, CT, United States

**Keywords:** Genetic animal models, Bone μCT, Bone histomorphometry, Osteocytes, Osteoclasts

## Abstract

Craniometaphyseal dysplasia (CMD), a rare craniotubular disorder, occurs in an autosomal dominant (AD) or autosomal recessive (AR) form. CMD is characterized by hyperostosis of craniofacial bones and flaring metaphyses of long bones. Many patients with CMD suffer from neurological symptoms. To date, the pathogenesis of CMD is not fully understood. Treatment is limited to decompression surgery. Here, we report a knock in (KI) mouse model for AR CMD carrying a R239Q mutation in CX43. *Cx43*^*KI/KI*^ mice replicate many features of AR CMD in craniofacial and long bones. In contrast to *Cx43*^+/+^ littermates, *Cx43*^*KI/KI*^ mice exhibit periosteal bone deposition and increased osteoclast (OC) numbers in the endosteum of long bones, leading to an expanded bone marrow cavity and increased cortical bone thickness. Although formation of *Cx43*^+/+^ and *Cx43*^*KI/KI*^ resting OCs are comparable, on bone chips the actively resorbing *Cx43*^*KI/KI*^ OCs resorb less bone. Cortical bones of *Cx43*^*KI/KI*^ mice have an increase in degenerating osteocytes and empty lacunae. Osteocyte dendrite formation is decreased with reduced expression levels of *Fgf23*, *Sost*, *Tnf-α*, *IL-1β*, *Esr1, Esr2*, and a lower *Rankl/Opg* ratio. Female *Cx43*^*KI/KI*^ mice display a more severe phenotype. Sexual dimorphism in bone becomes more evident as mice age. Our data show that the CX43_R239Q_ mutation results in mislocalization of CX43 protein and impairment of gap junction and hemichannel activity. Different from CX43 ablation mouse models, the CX43_R239Q_ mutation leads to the AR CMD-like phenotype in *Cx43*^*KI/KI*^ mice not only by loss-of-function but also via a not yet revealed dominant function.

## Introduction

Craniometaphyseal dysplasia (CMD) is a rare genetic bone disorder characterized by progressive hyperostosis of craniofacial bones and flared metaphyses of long bones.^1^ Diagnosis of CMD is based on clinical findings, radiographic examination, and genetic mutations. Craniofacial features of patients with CMD include hypertelorism, widened nasal bridge, paranasal bossing, widely spaced eyes with increased zygomatic width, and prominent mandibles.^2^ Progressive thickening of craniofacial bones can lead to compression of cranial nerves resulting in neurological symptoms, including visual or hearing impairment, and facial palsy.^3–5^ Associated Chiari I malformation can result in severe headaches.^6^ CMD is often diagnosed early during infancy due to difficulties in breathing and feeding. Radiographic images show hyperostotic calvarial and facial bones, sclerotic skull base, and thickened cortical bones of all proximal phalanges.^3^ CMD occurs as an autosomal dominant (AD, OMIM 605145) trait with mutations in the progressive ankylosis (*ANKH*) gene and in an autosomal recessive form (AR, OMIM 121014) carrying a R239Q mutation in Connexin 43 (CX43), encoded by the gap junction protein alpha 1 (*GJA1*) gene.^7–9^ Laboratory findings include normal or transiently decreased blood calcium and phosphate, elevated serum alkaline phosphatase (ALP), normal parathyroid hormone (PTH).^10,11^ There are no specific biomarkers for CMD. To date, CMD is managed by decompression surgeries to relieve neurological symptoms. Effective pharmaceutical therapy for CMD is still missing largely because that the pathogenesis of CMD remains incompletely understood.

To study the pathogenesis of CMD, we have generated an AD CMD mouse model (*Ank*^*KI/KI*^ mice) expressing an ANK_F377del_ mutation.^12^ *Ank*^*KI/KI*^ mice mimic characteristic features, including hyperostosis of craniofacial bones and flaring metaphyses of long bones. Young *Ank*^*+/KI*^ mice are phenotypically similar to *Ank*^+/+^ mice but 1-year-old *Ank*^+*/KI*^ mice display an AD CMD-like phenotype. Our studies have shown that mutant ANK severely impairs OC formation and resorption via cytoskeleton disruption and by reducing the migration of OC progenitors.^13^ The pathogenesis of CMD is complex as CMD mutant ANK acts partially via loss-of-function due to rapid protein degradation but at the same time has gain-of-function properties via altered protein-protein interaction.^14^ It is still under investigation whether ANKH_F377del_ and CX43_R239Q_ mutations contribute to CMD via different mechanisms or whether there are any common targets involved. Compared to ANKH, the structure, function, effect on bone, regulation or downstream targets of CX43 has been more extensively studied. While *Ank*^*KI/KI*^ mice are a powerful tool, we propose that the generation of an AR CMD mouse model (*Cx43*^*KI/KI*^) by introducing the CX43_R239Q_ mutation greatly facilitates CMD research because more molecular tools for studying CX43 are available.

Connexins are encoded by 21 genes in humans and 20 genes in mice and are widely expressed in nearly all tissues.^15^ Each connexin has four transmembrane domains, two extracellular loops, one intracellular loop, and intracellular amino and carboxyl termini.^16^ Six connexins form hemichannels that may serve as pores or may join head to head with hemichannels in a neighboring cell to form gap junctions that allow exchange of small molecules (<1 kDa) and ions between adjacent cells.^17 18^ CX43 is the predominant gap junction detected in osteoblasts, osteoclasts, osteocytes, and chondrocytes, although CX45, CX46, CX26, and CX37 are also expressed in bone cells.^19–25^ Mutations in connexin genes are associated with a large variety of disorders affecting many organs.^26^ Specific to bone, mutations in CX43 are responsible for AR CMD and oculodentodigital dysplasia (ODDD), characterized by syndactyly, microphthalmia, craniofacial and dental abnormalities.^9,27,28^ CX43-related diseases suggest that other connexins cannot sufficiently compensate for mutated CX43, which has predominant effects on functions of bone cells.

The roles of CX43 in skeletal development and bone homeostasis have been demonstrated in multiple mouse models with CX43 deficiencies. Global *Cx43*-null mice exhibit delayed endochondral and intramembranous ossification in addition to neural crest cell migration defects.^29^ CX43 has been conditionally deleted in early osteochondro progenitors (*Dermo1-Cre;Cx43*^−*/fl*^ mice),^30,31^ osteoblasts (*ColI-Cre;Cx43*^−*/fl*^ mice),^32–34^ mature osteoblasts and osteocytes (*Ocn-Cre;Cx43*^−*/fl*^ mice; *Dmp1-Cre;Cx43*^*fl/fl*^ mice).^35–38^ Moreover, mice carrying different missense point mutations in *Gja1* have been generated to study ODDD.^39–41^ These *Cx43* mutant mice replicate human ODDD phenotypes including craniofacial bone anomalies, syndactyly, and enamel hypoplasia. ODDD mutations in CX43 result in reduced formation and function of CX43.^39–43^ Conditional CX43 knockout mouse studies have shown that CX43 affects mineral density, geometrical, and biomechanical properties of the skeleton as well as bone cell response to fracture and mechanotransduction by acting on osteoblasts, osteoclasts, and osteocytes, however, some results remain controversial.^30–38^

Our group identified a novel missense mutation (c.716G>A, p.Arg239Gln), located in the C-terminus of *GJA1*, in six patients with AR CMD by whole-exome sequencing.^9^ We introduced the CX43_R239Q_ mutation in mice by CRISPR/Cas9 technology. *Cx43*^*KI/KI*^ mice display many features of AR CMD and do not completely phenocopy *Ank*^*KI/KI*^ mice, a mouse model for AD CMD. *Cx43*^*KI/KI*^ mice also exhibit the unique bone remodeling features that have not been reported in other global or conditional *Cx43* knockout mice. Interestingly, sex dimorphism in bone is observed and becomes more evident in aged female *Cx43*^*KI/KI*^ mice. This model, together with the AD CMD model, can be used to elucidate how skeletal development, remodeling, and metabolism are altered in CMD.

## Results

### Generation and characterization of CX43_R239Q_ KI mice, a model for AR CMD

We introduced a CX43_R239Q_ mutation identified in AR CMD into the genome of C57Bl/6J mice using CRISPR/Cas9 mediated gene editing. *Cx43*^*KI/KI*^ mice were viable and fertile. The ratio of *Cx43*^+/+^, *Cx43*^+*/KI*^ and *Cx43*^*KI/KI*^ litters followed Mendelian distribution (*Cx43*^+/+^: *Cx43*^+*/KI*^: *Cx43*^*KI/KI*^ = 64: 96: 58, n=218). Approximately 40% of *Cx43*^+*/KI*^ breeder pairs lost some pups during nursing, likely due to inappropriate feeding capability indicated by lack of milk spots in some mice (Fig. S1a). *Cx43*^+/+^ and *Cx43*^*KI/KI*^ mice were indistinguishable at birth and had comparable weight gain between 3–12 weeks of age (Fig. S1b). *Cx43*^*KI/KI*^ mice showed significantly increased femur length compared to wild type littermates (Fig. S1c).

**Figure 1:**
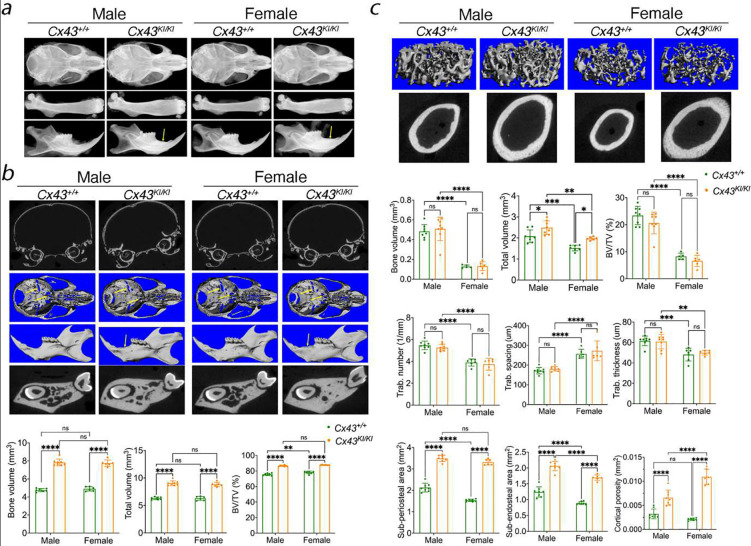
Skeletal analysis of male and female *Cx43*^+/+^ and *Cx43*^*KI/KI*^ mice at age of 3 months. **a**) Representative radiographic images of skulls, femurs, and mandibles; Representative μCT images of **b**) Cross-sections of calvariae, 3D images of cranial base, mandibles, and cross-sections of mandibles through the furcation of 1^st^ molar; yellow arrows indicate thickened mandibular alveolar bone and cranial foramina, which are narrowed in *Cx43*^*KI/KI*^ mice. **c**) Trabecular bone in metaphyses and cortical bone in the mid-diaphyses. Histograms shows quantitative measurements of bone parameters by μCT analysis. Statistics were performed by two-way ANOVA followed by Tukey’s post-hoc test (* *p*<0.05, ** *p*<0.01, *** and **** *p*<0.001). Data presented = mean ± SD.

To examine whether *Cx43*^*KI/KI*^ mice replicate CMD-like skeletal phenotypes, we first performed radiographic imaging and μCT analysis in *Cx43*^+/+^ and *Cx43*^*KI/KI*^ male and female mice at age of 3 months. *Cx43*^*KI/KI*^ mice had increased radiopacity of craniofacial bones, club-shaped femurs with thickened diaphyseal cortical bone, and increased alveolar bone mass with normal tooth eruption and positioning of cervical loops (Fig. 1a). μCT analysis showed that *Cx43*^*KI/KI*^ mice exhibited thickening of skull bones, narrowed neural foramina at the cranial base, increased bone volume (BV), total volume (TV), and BV/TV in jawbones (Fig. 1b). *Cx43*^*KI/KI*^ mice displayed significantly increased periosteal and endosteal perimeter, increased cortical diaphyseal porosity, as well as increased cortical thickness (*Cx43*^+/+^: *Cx43*^*KI/KI*^ male mice = 0.18±0.01: 0.23±0.01, p<0.01; *Cx43*^+/+^: *Cx43*^*KI/KI*^ female mice = 0.15±0.01: 0.28±0.02, p<0.01) (Fig. 1c). *Cx43*^+/+^ and *Cx43*^*KI/KI*^ mice did not significantly differ in metaphyseal measurements like trabecular bone mass, trabecular number, trabecular spacing, and trabecular thickness but *Cx43*^*KI/KI*^ mice showed increased total volume indicating widened metaphyses (Fig. 1c). There were no significant differences in vertebrae in *Cx43*^+/+^ and *Cx43*^*KI/KI*^ mice (Fig. S2). While both sexes exhibited skeletal abnormalities, female *Cx43*^*KI/KI*^ mice presented with a more prominent phenotype. Taken together, these data confirmed that *Cx43*^*KI/KI*^ mice replicated many features of AR CMD patients, including skull and jawbone thickening, narrowed neural foramina of the cranial base, and widened metaphyses with hypersclerotic diaphyseal cortical bone in femurs.

### Effects of CX43_R239Q_ mutation on bone turnover in *Cx43*^*KI/KI*^ mice

**Figure 2:**
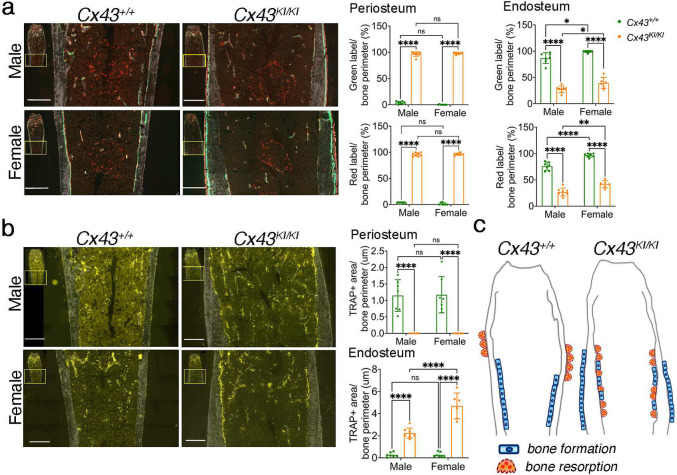
Static and dynamic histomorphometry of femoral cortical bones in male and female *Cx43*^+/+^ and *Cx43*^*KI/KI*^ mice at age of 3 months. **a**) Representative images showing calcein (green) and alizarin complexone (red) double staining. Histograms show quantitative measurements of labeling on periosteum and endosteum; **b**) Representative images showing TRAP staining. Histograms showed quantitative measurements of TRAP positive stained area on periosteum and endosteum; **c**) Schematic summarizing the different patterns of bone formation and resorption activities in *Cx43*^+/+^ and *Cx43*^*KI/KI*^ mice. Statistics were performed by two-way ANOVA followed by Tukey’s post-hoc test. Scale bar = 500 μm. Data presented = mean ± SD (* *p*<0.05, ** *p*<0.01, *** and **** *p*<0.001).

To dissect out the effects of CMD-mutant CX43 on bone turnover, we first performed static and dynamic histomorphometry in femurs of 3-month-old *Cx43*^+/+^ and *Cx43*^*KI/KI*^ male and female mice. Consistent with findings from μCT, the CX43_R239Q_ mutation affects turnover in cortical bone more than in metaphyseal trabeculation. There were no significant differences in mineral apposition rate (MAR), bone formation rate (BFR), osteoblast surface (AP/BS) including bone forming (AP_L/BS) and bone lining surfaces (AP_NL/BS), osteoclast surface (TRAP/BS) including bone resorbing surface (TRAP_L/BS) and bone remodeling surface (TRAP_NL/BS) in femoral metaphyses of *Cx43*^+/+^ and *Cx43*^*KI/KI*^ mice (Table 1). In general, female mice exhibited increased osteoclast surface (TRAP/BS) and bone remodeling unit surface (AP_TRAP_R/BS) than male mice (Table 1). On the other hand, diaphyseal cortical bones of *Cx43*^+/+^ and *Cx43*^*KI/KI*^ mice showed very different patterns. Active bone formation, suggested by the presence of calcein (green) and alizarin complexone (red) labeling, was observed on the periosteal surface of *Cx43*^*KI/KI*^ mice but mostly on the endosteal surface of *Cx43*^+/+^ mice (Fig. 2a). We next analyzed TRAP-positive cells, indicative of bone resorption activity, in cortical bones in the mid-diaphyseal region of femurs. While *Cx43*^+/+^ mice showed some TRAP staining on the periosteal surface, mostly below the widest portion of metaphyses, *Cx43*^*KI/KI*^ exhibited significantly increased TRAP-positive cells on the endosteal surface and very little on periosteum (Fig. 2b). In comparison to male *Cx43*^*KI/KI*^ mice, the interlabel distance in cortical bone was increased in female *Cx43*^*KI/KI*^ mice (male *Cx43*^*KI/KI*^ mice: female *Cx43*^*KI/KI*^ mice = 54.44 ± 10.93: 104.7 ± 13.43 μm, p < 0.001 by Student’s *t*-test). Our data, summarized in Fig. 2c, showed that *Cx43*^*KI/KI*^ mice have increased periosteal bone formation and endosteal bone resorption whereas *Cx43*^+/+^ mice mostly exhibited endosteal bone formation and periosteal resorption. This abnormal pattern of bone formation and resorption results in cortical bone thickening in the diaphysis and an enlarged bone marrow cavity with club-shaped femurs in *Cx43*^*KI/KI*^ mice.

**Figure 3:**
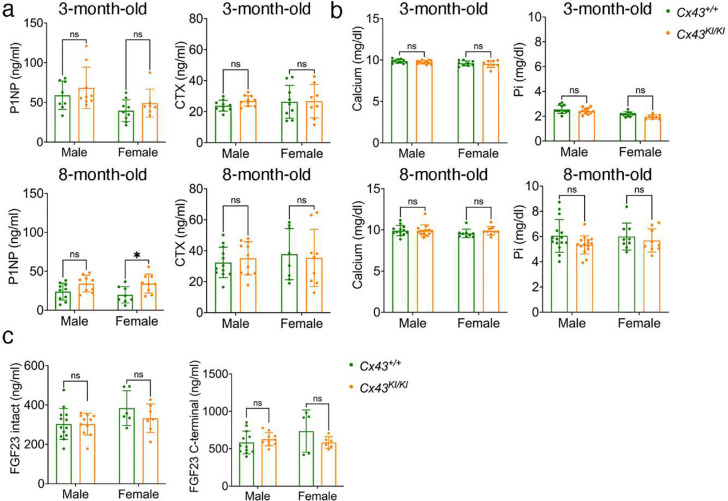
Biochemical analysis of male and female *Cx43*^+/+^ and *Cx43*^*KI/KI*^ mice at ages of 3 and 8 months. **a**) Serum levels of P1NP, a marker for bone formation, and CTX, a marker for bone resorption; **b**) Serum levels of Ca and Pi; and **c**) Serum levels of intact (active) and C-terminal form (inactive) of FGF23 at 8-month-old. Statistics were performed by two-way ANOVA followed by Tukey’s post-hoc test (* *p*<0.05).

We next measured serum levels of P1NP, a marker for bone formation, and CTX, a marker for bone resorption, in male and female *Cx43*^+/+^ and *Cx43*^*KI/KI*^ mice at ages of 3 and 8 months. Serum levels of P1NP were similar in 3-month-old *Cx43*^+/+^ and *Cx43*^*KI/KI*^ mice but were significantly increased in 8-month-old female *Cx43*^*KI/KI*^ mice when compared to female *Cx43*^+/+^ mice of the same age (Fig. 3a). Serum levels of CTX remained comparable between *Cx43*^+/+^ and *Cx43*^*KI/KI*^ mice at ages of 3 and 8 months (Fig. 3a). 8-month-old mice showed remarkable reduction of P1NP levels and elevation of CTX levels compared to 3-month-old mice, suggesting somewhat decreased bone formation and increased bone resorption with aging. Our data showed that circulating P1NP and CTX levels do not reflect the tissue-specific differences in bone formation and resorption in *Cx43*^+/+^ and *Cx43*^*KI/KI*^ mice. Serum calcium (Ca) and phosphate (Pi) levels were reported to be within normal range or slightly decreased in patients with AR CMD.^9,44^ Serum Ca and Pi levels in *Cx43*^+/+^ and *Cx43*^*KI/KI*^ mice were comparable, both at ages of 3 and 8 months (Fig. 3b). We then measured fibroblast growth factor 23 (FGF23) levels, a phosphaturic factor secreted by bone to promote Pi wasting in kidney. We previously reported that the active form of FGF23 (intact FGF23) in *Ank*^*KI/KI*^ mice, a model for AD CMD, was comparable but found an increased inactive form of FGF23 (C-terminal FGF23).^10^ Our data showed that there were no significant differences in intact FGF23 and C-terminal FGF23 levels between *Cx43*^+/+^ and *Cx43*^*KI/KI*^ mice (Fig. 3c).

### Progressive worsening of bone phenotype as *Cx43*^*KI/KI*^ mice age

**Figure 4:**
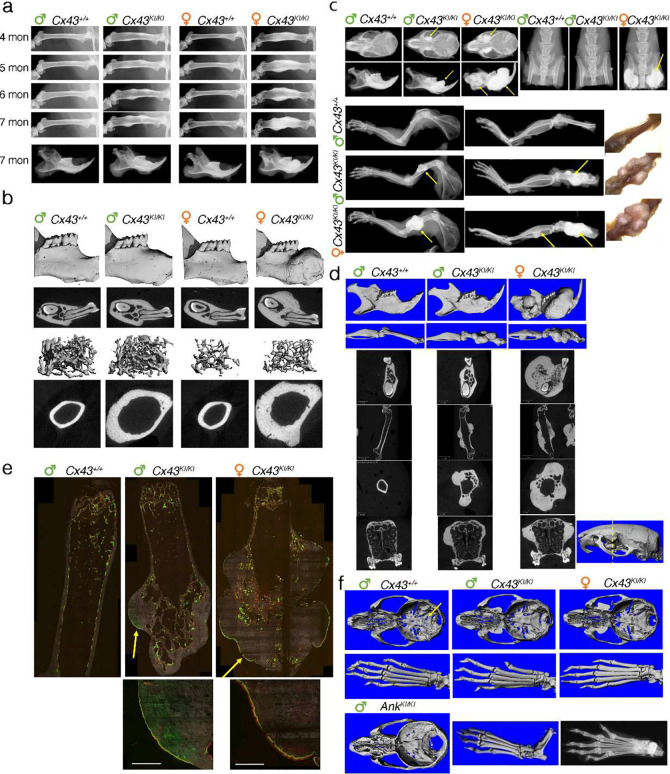
Skeletal analysis of male and female *Cx43*^+/+^ and *Cx43*^*KI/KI*^ aged mice. **a**) Representative radiographs of femurs and mandibles from 4- to 7-month-old mice; **b**) Representative μCT images of 3D mandibles, cross-sections of mandibles through the furcation of 1^st^ molar, femoral trabecular and cortical bones from 7-month-old mice; **c**) Radiographs of skulls, mandibles, forearms, legs from 1-year-old *Cx43*^+/+^ and *Cx43*^*KI/KI*^ mice; photos showing femoral bones; **d**) Representative μCT 3D images of mandibles and femurs, 2D images of cross-sections of mandibles, femurs (longitudinal and cross sections), and skull (cross section through the yellow dotted line) from 1-year-old *Cx43*^+/+^ and *Cx43*^*KI/KI*^ mice; **e**) Dynamic histomorphometry of 1-year-old *Cx43*^+/+^ and *Cx43*^*KI/KI*^ mice; **f**) Cranial neural foramina and joint phenotype from 1-year-old *Cx43*^+/+^, *Cx43*^*KI/KI*^ and 3-month-old *Ank*^*KI/KI*^ mice.

We next analyzed skeletal phenotypes in aging mice since CMD progresses throughout life. Male and female *Cx43*^*KI/KI*^ mice progressively developed bone outgrowths in multiple sites of craniofacial and long bones, including but not limited to femurs and mandibles, shown by radiographs taken at ages of 4, 5, 6, and 7 months (Fig. 4a, data not shown). Consistent with radiographs, μCT analysis of 7-month-old bones showed grossly increased bone masses in mandibles and femurs (Fig. 4b). Sexual dimorphism became more evident with aging. At one-year-old, female *Cx43*^*KI/KI*^ mice had significantly more prominent bone masses at multiple sites compared to male *Cx43*^*KI/KI*^ mice (Fig. 4c–4d). Interestingly, increased interlabel distance in 3-month-old female *Cx43*^*KI/KI*^ mice continued to be observed in 1-year-old mice, suggesting more new bone deposition in female than male *Cx43*^*KI/KI*^ mice (Fig. 4e). When compared to 3-month-old AD CMD *Ank*^*KI/KI*^ mice, 1-year-old *Cx43*^*KI/KI*^ mice show less severe narrowing of cranial foramina and had no joint stiffness phenotype (Fig. 4f). In summary, our data showed progressive worsening of bone thickening and sexual dimorphism in skeletal abnormalities in *Cx43*^*KI/KI*^ mice.

### Effect of CX43_R239Q_ on osteoblast and osteoclast cultures

**Figure 5:**
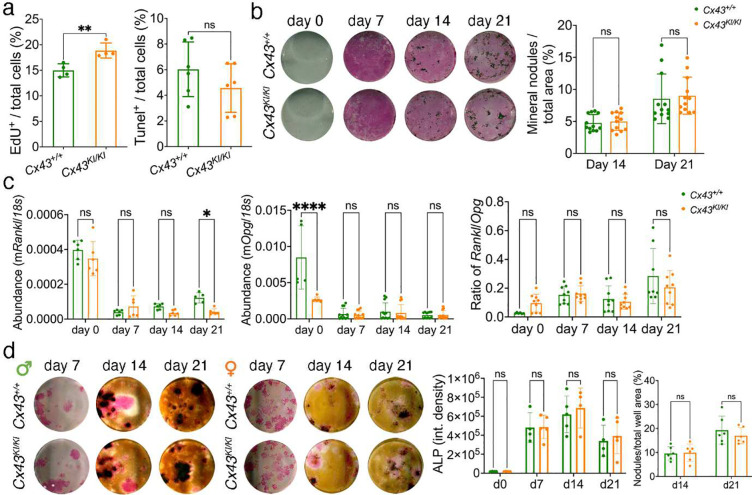
Osteoblast cultures of *Cx43*^+/+^ and *Cx43*^*KI/KI*^ mice. **a**) Cell proliferation and apoptosis in mCOB cultures analyzed by EdU and Tunel assays, respectively; **b**) Alkaline phosphatase (ALP) and mineral nodule formation by von Kossa staining; **c**) *Rankl* and *Opg* mRNA expression levels in mCOB cultures; **d**) ALP and mineral nodule formation in bone marrow stromal cultures (BMSCs). Histograms showing no significant differences in male mice. Statistical analysis was performed by two-way ANOVA followed by Tukey’s post-hoc test.

**Figure 6:**
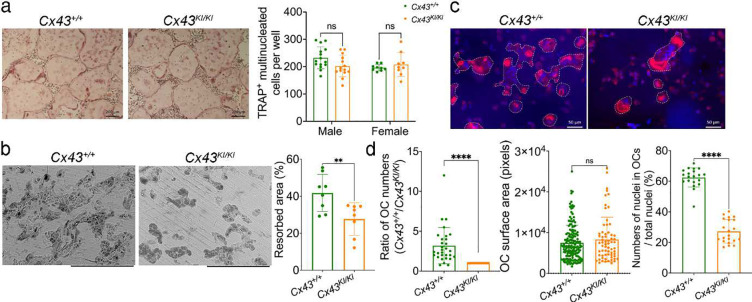
Cx43_R239Q_ mutational effects on osteoclasts (OCs). **a**) Formation of resting OCs (rOCs) was comparable between *Cx43*^+/+^ and *Cx43*^*KI/KI*^ BMM cultures; scale bar = 200 μm. **b**) Resorption on bone chips by actively resorbing *Cx43*^*KI/KI*^ OCs (aOCs) was reduced; scale bar = 300 μm. **c**) *Cx43*^+/+^ and *Cx43*^*KI/KI*^ aOCs stained by rhodamine phalloidin; scale bar = 50 μm. **d**) Quantitative measurements of aOCs numbers that formed, surface area of aOCs, and aOC fusion, which is measured by nuclei numbers in aOCs divided by total nuclei numbers on bone chips. Statistical analysis was performed by Student’s *t*-test (* *p*<0.05, ** *p*< 0.01, **** *p*<0.001).

To examine mutational effects of CX43_R239Q_ on osteoblasts, we isolated calvarial osteoblasts (mCOBs) and bone marrow stromal cells (BMSCs) from *Cx43*^+/+^ and *Cx43*^*KI/KI*^ mice and cultured cells in osteogenic differentiation medium for 21 days. We examined cell proliferation and apoptosis 2 days after plating mCOBs by EdU and Tunel assays, respectively. *Cx43*^*KI/KI*^ mCOBs displayed significantly increased proliferation but comparable apoptosis in comparison to *Cx43*^+/+^ mCOBs (Fig. 5a). Mineral nodule formation of mCOBs cultured in osteogenic medium for 14 and 21 days were comparable between *Cx43*^+/+^ and *Cx43*^*KI/KI*^ mice (Fig. 5b). Expression levels of osteoblast marker genes examined by qPCR including *ColI*, *Alp*, *Opn*, *Osx*, *Runx2*, *Ocn* were comparable between *Cx43*^+/+^ and *Cx43*^*KI/KI*^ mCOBs (Fig. S3a). However, *Opg* at day 0 was increased and *Rankl* at day 21 was decreased in *Cx43*^*KI/KI*^ mCOBs, although the *Rankl/Opg* ratio was not significantly different for any time points (Fig. 5c). Similarly, ALP staining and mineral nodule formation were not significantly different in *Cx43*^+/+^ and *Cx43*^*KI/KI*^ BMSCs from male and female mice (Fig. 5d). These data are consistent with histomorphometry data that show no significant difference in OB activity in the metaphyseal region of femurs in *Cx43*^+/+^ and *Cx43*^*KI/KI*^ mice. Data obtained from mCOB or BMSC cultures, however, do not reflect the increased bone formation in the periosteum of *Cx43*^*KI/KI*^ femurs shown by dynamic histomorphometry described above. To examine the Cx43_R239Q_ mutational effects on OCs, we first cultured bone marrow-derived macrophages (BMMs) on culture plates and induced the formation of resting osteoclasts (rOCs) by supplementation with M-CSF and RANKL. TRAP+ cells with more than 3 nuclei were counted as OCs. There was no significant difference in the formation of rOCs between *Cx43*^+/+^ and *Cx43*^*KI/KI*^ cultures (Fig. 6a). Expression levels of OC marker genes that included *Nfatc1*, *Rank*, *Cathepsin K*, *αv integrin, β3 integrin,* and *Mmp9* were comparable between *Cx43*^+/+^ and *Cx43*^*KI/KI*^ rOCs (Fig. S3b). We next plated BMMs on bone chips to examine the formation and function of actively resorbing osteoclasts (aOCs). Interestingly, the bone resorption pit assay showed a significant reduction of the resorptive activity of *Cx43*^*KI/KI*^ aOCs when compared to *Cx43*^+/+^ aOCs (Fig. 6b). We next examined the aOCs by rhodamine phalloidin staining and found that reduced numbers of aOCs formed on bone chips (Fig. 6c & 6d). The size of *Cx43*^+/+^ and *Cx43*^*KI/KI*^ aOCs were not significantly different but there was decreased fusion activity in *Cx43*^*KI/KI*^ aOCs, suggested by reduced numbers of nuclei in OCs normalized to total nuclei numbers (Fig. 6d).

### Effect of CX43_R239Q_ on osteocytes

**Figure 7:**
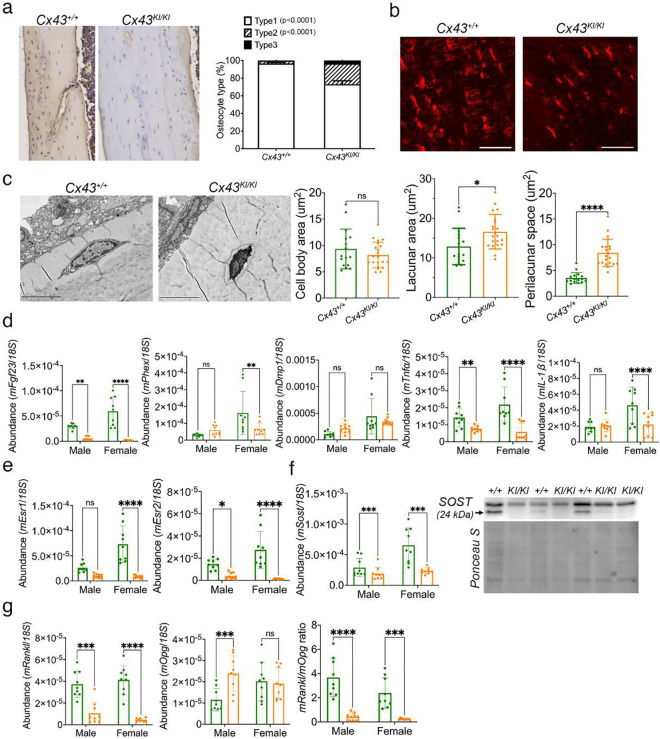
Osteocyte phenotype in *Cx43*^*KI/KI*^ mice. **a**) Diaphyseal cortical bone showing increased numbers of degenerating osteocytes and empty lacunae in *Cx43*^*KI/KI*^ mice; **b**) Dendrite formation of osteocytes stained by rhodamine phalloidin; scale bar = 50 μm; **c**) Osteocytes imaged by scanning electron microscope (SEM) and quantitative measurements of areas of osteocyte bodies, lacunar area, and perilacunar space; (scale bar = 5 μm; statistical analysis was performed by Student’s *t*-test (ns: no significant difference, * *p*<0.05, **** *p*<0.001)). **d**) Expression levels of *Fgf23*, *Phex*, *Dmp1*, *Tnf*α, and *IL-1*β by qPCR; **e**) mRNA levels of *Esr1* and *Esr2*; **f**) *Sost* mRNA and protein levels; **g**) *Rankl* and *Opg* mRNA levels. The ratio of *Rankl/Opg* is decreased in *Cx43*^*KI/KI*^ mice. Statistical analysis was performed by two-way ANOVA followed by Tukey’s post-hoc test (* *p*<0.05, ** *p*<0.01, *** and **** *p*<0.001).

Osteocytes are the most abundant cell type in bone and regulate the quality of bone matrix by directing osteoblasts and osteoclasts via dendrites and secreted molecules. To examine the effects of CX43_R239Q_ on osteocytes, we examined osteocytes in diaphyseal cortical bone. We categorized osteocytes into three types: type I, viable osteocytes; type II, degenerating osteocytes; and type III, empty lacunae (Fig. S4). While the number of type II and III osteocytes were significantly increased, type I osteocytes were less in *Cx43*^*KI/KI*^ mice (Fig. 7a). The dendrites of *Cx43*^*KI/KI*^ osteocytes were reduced in number and length shown by rhodamine phalloidin staining (Fig. 7b). We next visualized osteocytes by scanning electron microscopy. The lacunar area of *Cx43*^*KI/KI*^ osteocytes was significantly increased due to increased perilacunar space but the cell body area was comparable between *Cx43*^+/+^ and *Cx43*^*KI/KI*^ osteocytes (Fig. 7c). Expression levels of *Fgf23* and *Phex* in the cortex of femurs were significantly decreased in *Cx43*^*KI/KI*^ bones while *Dmp1* was comparable to *Cx43*^+/+^ bones (Fig. 7d). Osteocytes can express pro-inflammatory cytokines such as *Tnf-*α, and *IL-1β*, which can lead to increased osteoclastogenesis and inhibit osteoblast formation.^45–47^ *Tnf-α* and *IL-1*β were also significantly reduced in *Cx43*^*KI/KI*^ bones (Fig. 7d). Female *Cx43*^*KI/KI*^ mice displayed a stronger bone phenotype than male *Cx43*^*KI/KI*^ mice. Therefore, we examined expression levels of *Esr1* and *Esr2* encoding estrogen receptor α and β. *Esr2* was significantly decreased in both male and female *Cx43*^*KI/KI*^ bones while *Esr1* was only negatively affected in female *Cx43*^*KI/KI*^ bones (Fig. 7e). Osteocytes can express *Sost* to inhibit bone formation and *Rankl* to stimulate osteoclast activities.^48,49^ *Sost* mRNA and protein levels were significantly decreased and the *Rankl/Opg* ratio was reduced in *Cx43*^*KI/KI*^ bones, suggesting that the increased bone mass in *Cx43*^*KI/KI*^ mice may be due to osteocyte-promoted bone formation and osteocyte-mediated inhibition of bone resorption (Fig. 7f & 7g).

### Expression and function of Cx43_R239Q_ mutant protein

**Figure 8:**
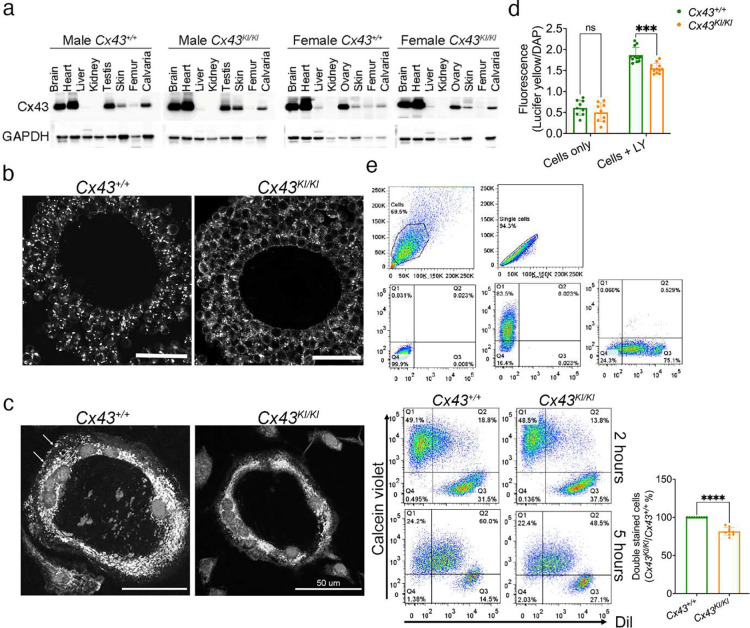
Expression, localization, and function of mutant *Cx43*^*KI/KI*^ protein. **a**) Tissue expression of wild type and mutant CX43 by immunoblotting; **b**) Localization of wild type and mutant Cx43 in ovaries; scale bar = 50 μm; **c**) Localization of wild type and mutant CX43 in OCs; scale bar = 50 μm; **d**) Hemichannel activity; statistical analysis performed by two-way ANOVA followed by Tukey’s post-hoc test (ns: no significant difference, *** *p* < 0.001). **e**) Gap junction activity by flow cytometry. Forward and side scatter has been applied to select the single-cell population and to remove debris (top panel). The gating strategies are defined by parameters obtained from cells with no staining and single staining (Calcein violet or Dil) (second panel from the top). Double stained cells (Q2) in *Cx43*^+/+^ and *Cx43*^*KI/KI*^ groups were quantified in the histogram. Statistical analysis was performed by Student’s *t*-test (ns: no significant difference, **** *p* < 0.001).

We found CX43 and CX43_R239Q_ protein most abundantly expressed in brain, heart, testis/ovary and to a lesser amount in skin, bone, and liver but were undetectable in kidney (Fig. 8a). Expression levels of *Cx43* mRNA during osteoblastogenesis and osteoclastogenesis were comparable between *Cx43*^+/+^ and *Cx43*^*KI/KI*^ mCOB/BMSC and BMM cultures, respectively (data not shown). We next examined the localization of wt and mutant CX43 protein in ovaries and multinucleated OCs. CX43 immunostaining of ovaries showed large punctae associated with the plasma membrane in *Cx43*^+/+^ mice compared to a weaker staining and less concentrated distribution in *Cx43*^*KI/KI*^ mice (Fig. 8b). Similarly, CX43 was detected on the plasma membrane in *Cx43*^+/+^ OCs while expression of mutant CX43 was mostly inside the cells in *Cx43*^*KI/KI*^ OCs (Fig. 8c). CX43 serves as a hemichannel and gap junction protein when located on the plasma membrane. To examine whether the hemichannel function is compromised, skin fibroblasts isolated from *Cx43*^+/+^ and *Cx43*^*KI/KI*^ mice were loaded with Lucifer Yellow dye and the amount of dye transfer was measured by a microplate reader. Dye transfer was reduced in *Cx43*^*KI/KI*^ cells compared to *Cx43*^+/+^ cells (Fig. 8d). Furthermore, the gap junction activity measured by a parachute assay with DilC_18_ dye (recipient cells) and calcein violet AM dye (donor cells) also showed reduced activity in *Cx43*^*KI/KI*^ cells (Fig. 8e). In summary, the CX43_R239Q_ mutation does not affect expression levels but results in the mislocalization of mutant CX43 protein leading to the compromised hemichannel and gap junction function.

## Discussion

AD CMD is more common than the AR form. Prior to the identification of the CX43_R239Q_ mutation for AR CMD,^9^ there were a small number of presumed AR CMD cases reported without genetic validation.^50–53^ There has been a tendency, although inconsistent, to consider more severe CMD cases as AR CMD.^52,54^ Characteristics common to AD and AR CMD include hyperostosis of craniofacial bones, flaring of metaphyses, widened nasal bridge, prominence of the frontal bones, hypertelorism, and mandibular prognathism. Patients with AR CMD have relatively more hypersclerotic diaphyseal cortical bones but less severe cranial neural deficits.^51^ Most reported patients with AR CMD have normal to mild vision or hearing impairment while most AD CMD patients have cranial nerve compression with some degree of facial paralysis, blindness, deafness, or severe headache. We have successfully generated a *Cx43*^*KI/KI*^ mouse model for AR CMD. *Cx43*^*KI/KI*^ mice exhibit skeletal anomalies including hyperostotic craniofacial bones and widened metaphyses with hypersclerotic cortical bones. We previously reported a mouse model for AD CMD (*Ank*^*KI/KI*^ mice) carrying an *ANK*_*F377del*_ mutation. *Ank*^*KI/KI*^ mice show a CMD-like phenotype at birth and *Ank*^*+/KI*^ mice display a milder phenotype approximately at 1-year-old.^12^ When comparing *Cx43*^*KI/KI*^ mice to *Ank*^*KI/KI*^ mice, we noticed that: 1) *Cx43*^*KI/KI*^ mice were viable whereas *Ank*^*KI/KI*^ mice die around 5–6 months; 2) *Cx43*^*KI/KI*^ mice are fertile whereas *Ank*^*KI/KI*^ mice are infertile; 3) *Cx43*^*KI/KI*^ mice do not have joint stiffness as seen in *Ank*^*KI/KI*^ mice. Joint stiffness is absent in *Ank*^+*/KI*^ mice and patients with AD CMD; 4) *Cx43*^*KI/KI*^ mice develop more hypersclerotic diaphyseal cortical bone than *Ank*^*KI/KI*^ mice; 5) *Cx43*^*KI/KI*^ mice have milder narrowing of cranial foramina than *Ank*^*KI/KI*^ mice; 6) Only *Cx43*^*KI/KI*^ mice progressively develop irregular bone bossing at multiple sites. Localized new bone on the shaft of fibulae has been reported in a patient with AR CMD.^52^ Excessive craniofacial bone bossing was reported in a patient with atypical CMD.^55^

AD and AR CMD are caused, in part of, by loss-of-function of ANKH and CX43, respectively, utilizing different mechanisms. The F377del mutation leads to rapid protein degradation of ANK resulting in significantly reduced ANK_F377del_ protein.^14^ The R239Q mutation in CX43, on the other hand, does not affect protein expression levels. Loss-of-function of CX43_R239Q_ is caused by the mislocalization of mutant CX43. Our future study will focus on whether the CX43 mutation affects the trafficking of CX43 and CX43 binding protein partners. The R239Q mutation is located at the CX43 intracellular C-terminal domain, which may interact with various protein partners, including molecules involved in adherens junctions such as the cadherins, α- and β-catenin; kinases and phosphatases that regulate the assembly or function of CX43, and with cytoskeletal proteins such as actin, actin-binding protein, and microtubules. The R239Q mutation is within the tubulin binding motif (residues 228–260).^56–58^ Interaction between CX43 and tubulin/microtubules can affect the intracellular trafficking of CX43, regulate gap junction function, and control the TGF-β pathway via competing with Smad2 for tubulin/microtubule binding.^57^

Mice with global CX43 deletion (*Cx43*^*KO/KO*^ mice) die within one hour of delivery due to heart anomalies and muscular contractions.^29^ These *Cx43*^*KO/KO*^ mice have delayed intramembranous and endochondral ossification and impaired mineralization suggesting that CX43 is important for OB differentiation.^29^ Because of the perinatal lethality of a global *Cx43* deletion, conditional *Cx43* ablation models using the Cre/LoxP system in early osteochondro progenitors (*DM1Cre;Cx43*^−*/fl*^), osteoblasts (*ColICre; Cx43*^−*/fl*^), mature osteoblasts/osteocytes (*OcnCre;Cx43*^−*/fl*^), and osteocytes (*Dmp1Cre;Cx43*^*fl/fl*^) have been developed.^30–38^ These CX43 mouse models and our *Cx43*^*KI/KI*^ mice display a stronger phenotype in cortical bones than in cancellous bones, which may be explained by relatively low level of *Gja1* expression in trabecular bones and growth plates.^30^ Osteocyte apoptosis caused by lack of CX43 in mature osteoblasts/osteocytes is more pronounced in cortical bones.^37^ Our *Cx43*^*KI/KI*^ moue model differs from CX43 ablation models in several features. While *DM1Cre;Cx43*^−*/fl*^ and *ColICre; Cx43*^−*/fl*^ mice are osteopenic and *OcnCre;Cx43*^−*/fl*^ mice have no bone mass abnormalities,^30,32,35^ our *Cx43*^*KI/KI*^ mice display increased bone mass. Furthermore, conditional CX43 deletion mice have a thin cortex^30,33,38^ whereas our *Cx43*^*KI/KI*^ mice exhibit cortical bone thickening. While deletion of CX43 leads to an increased *Rankl/Opg* ratio,^30,36,37^ our *Cx43*^*KI/KI*^ mice show a decreased *Rankl/Opg* ratio. CTX, a serum marker for bone resorption, is increased in *DM1Cre;Cx43*^−*/fl*^ and *OcnCre;Cx43*^−*/fl*^ mice^30,36^ but is not altered in *Cx43*^*KI/KI*^ mice. mCOBs and BMSCs from *Cx43*^+/+^ and *Cx43*^*KI/KI*^ mice have comparable ALP and mineral nodule formation. This is in contrast to the reduced ALP and mineralization in osteoblast cultures with CX43 global deletion (*Cx43*^−/−^) and conditional CX43 ablation (*ColCre;Cx42*^−*/fl*^).^29,32^ In addition, the excessive bone bossing is only presented in the *Cx43*^*KI/KI*^ mice. Based on the different phenotypes of *Cx43*^*KI/KI*^ and *Cx43*^*KO/KO*^ mice we propose that AR CMD is not merely caused by loss-of-function.

The club-shaped femurs in *Cx43*^*KI/KI*^ mice are caused by an unusual pattern of periosteal bone formation and endosteal bone resorption. In *Cx43*^*KI/KI*^ mice we suspect that osteocytes dysregulate periosteal and endosteal bone cell activities. Osteocytes control the bone quality/strength by secretion of paracrine factors, mechanosensing, and maintaining mineral homeostasis via their lacunar-canalicular network. Dendrite defects have been associated with several skeletal diseases like osteoporosis, osteoarthritis, osteogenesis imperfecta.^59–61^ Osteocytes control bone remodeling by sending signals to osteoblasts and osteoclasts. For instance, osteocytes can produce sclerostin (*Sost*), which inhibits bone formation and receptor activator of nuclear factor-κB ligand (*Rankl*), which promotes bone resorption.^49,62^ Osteocytes also express *Fgf23,* which is linked to hypophosphatemia and impaired bone mineralization in autosomal-dominant hypophsophatemic rickets/osteomalacia (ADHR), X-linked hypophosphatemic rickets/osteomalcia (XLH), and tumor-induced osteomalacia (TIO).^63–65^ The proinflammatory cytokines tumor necrosis factor alpha (*Tnf-*α) and interleukin 1β (*IL-1β*) expressed by osteocytes can upregulate *Fgf23*.^66^ Estrogen signaling may contribute to sexual dimorphism in bone.^67^ Our data show that ER-α is only decreased in female *Cx43*^*KI/KI*^ mice while ER-β is reduced in both male and female *Cx43*^*KI/KI*^ mice. ER-α and ER-β are expressed in bone but regulate bone mass differently in male and female mice.^68–70^ Bone remodeling in male mice is regulated only by ER-α, whereas bone turnover in female mice is controlled by ER- α and ER-β.^71,72^ Interestingly, the serum estradiol level is significantly increased in female but not male ER-α−/− mice.^72^ In female ER-β−/− mice, the bone mineral content is increased due to increased cortical bone area.^73^ The reduced expression of ER-α and ER-β may partially explain the increased severity of bone mass in female *Cx43*^*KI/KI*^ mice.

In conclusion, our study presents a valid mouse model for investigating the molecular mechanisms of AR CMD. The R239Q mutation in CX43 affects osteoblasts, osteoclasts, and osteocytes, collectively contributing to the AR CMD-like skeletal phenotype. Intracellular mislocalization and differential gene expression provides insights into some of the bone abnormalities in *Cx43*^*KI/KI*^ mice. The unique skeletal features of *Cx43*^*KI/KI*^ mice suggest a yet unknown dominant function of CX43_R239Q_ that supplements the loss-of-function effects of CX43_R239Q_ in AR CMD.

## Materials and Methods

### Generation of the CX43_R239Q_ knockin (KI) mouse model and study approval

To generate CX43_R239Q_ KI mice (*Cx43*^*KI/KI*^), we used CRISPOR (http://crispor.tefor.net) to identify the *Cx43* target site, 5’-GGGCGTTAAGGATCGCGTGAAGG with the protospacer adjacent motif (PAM) for CRISPR/Cas9 mediated gene editing. *Cx43* sgRNA and Cas9 protein were mixed at room temperature for 15 min to form the RNP complex prior to adding *Cx43* R239Q ssDNA donor (5’-AT*T*G*AAGTAAGCATATTTTGGAGATCCGCAGTCTTTGGATGGGCTCAGTGGGCCGGTGGTGGCGTGGTAAGGATCGCTTCTTCCTTTCAC**CTG**ATCTTTAACGCCCTTGAAGAAGACATAGAAGAGCTCAATGATATTCAGAGCGAGAGAC*A*C*C-3’) for pronuclear microinjection into C57BL/6J one-cell embryos. The concentration of sgRNA, Cas9 and ssDNA donor was 50 ng, 200 ng and 50 ng per μL, respectively (all reagents from Integrated DNA Technologies, Coralville, IA).

Injected embryos were transferred into pseudo-pregnant females and potential founders were screened by PCR using primers (CxF1: 5’-GCTTCCTCTCACGTCCCACGGAG-3’ and CxR239QR: 5’- GGATCGCTTCTTCCTTTCACCT-3’) to amplify a fragment of 145 bp specific to the KI allele. Genotype of PCR-positive founders were further confirmed by PCR using primers (CxF1 and CxR1: 5’-GCTTGTTGTAATTGCGGCAGGAGG-3’) followed by sequencing of the PCR product (Fig. S5a and S5b). The R239Q mutation (CGC → CAG, pink bolded) and two silent mutations (blue bolded) were introduced to eliminate re-cleavage of the KI allele (Fig. S5a). A positive founder was bred with wildtype C57BL/6J mice (Stock number: 000664, The Jackson Laboratory). Mice were housed in an AAALAC-accredited facility under veterinary supervision. All experiments involving animals were in accordance with animal protocol AP-200644–0025 approved by the Animal Care Committee at the University of Connecticut Health (UConn Health).

### Skeletal analysis

Skulls, mandibles, and femurs of *Cx43*^+/+^ and *Cx43*^*KI/KI*^ male and female mice at ages of 3–12 months (n ≥ 5 for each group) were radiographed with a Kubtec Radiography System (KUB Technologies Inc., Stratford, CT) and analyzed by μCT (mCT20; ScanCo Medical AG, Bassersdorf, Switzerland) in the MicroCT facility at UConn Health as previously described.^74^ Mandibular data were collected by measuring vertical sections covering 1^st^ & 2^nd^ molars. Femoral trabecular data were taken at the distal growth plate over a distance of 960 μm. Cortical bone parameters were collected from 50 cross-sectional slices of 12 μm in the mid-diaphysis.

For the analysis of bone histomorphometry, we injected *Cx43*^+/+^ (n=8) and *Cx43*^*KI/KI*^ (n=8) male and female mice intraperitoneally with calcein (10 mg/kg body weight) and alizarin complexone (AC, 30 mg/kg body weight) at an interval of 7 days. Two days after the second injection, mice were sacrificed at 13 weeks of age and bones subjected to histomorphometry as previously described.^74^ Femurs were fixed in 10% formalin and frozen-embedded in OCT medium (Richard-Allan Scientific, San Diego, CA). Blocks were sectioned using a cryotome (CM3050S; Leica, Wetzlar, Germany). Three sections (7 μm thickness) revealing the central vein were collected using adhesive tape (Cryofilm type IIC; Section-Lab Co, Ltd., Yokohama, Japan).^74^ For dynamic histomorphometry, fluorescent images showing the mineralization status by calcein (green) and AC (red) were taken using a microscope slide scanner (Mirax Midi automated image acquisition system; Carl Zeiss, Jena, Germany). Parameters of bone forming activity were measured in the Computer Science Department at UConn.^74,75^

After scanning for mineralization imaging, the same sections were subjected to cellular staining. To detect OBs, ALP was stained by the fluorescent substrate fast red.^76^ AP-positive cells located on calcein- or AC-labeled bone surfaces were considered active osteoblastic cells whereas AP-positive cells on non-labeled bone surfaces were considered inactive bone lining cells.^74,75^ For OCs, tartrate resistant acid phosphatase (TRAP) staining was performed using a fluorescent substrate (ELF-97; Thermo Fisher Scientific, Waltham, MA). Bone surfaces that had both, mineralization labeling and TRAP signals, were considered remodeling surfaces whereas surfaces with TRAP-positive cells but no mineralization labeling were considered resorbing surfaces. The TRAP and AP signals were captured using filters optimized for tetracycline and tetramethyl rhodamine iso-thiocyanate (TRITC), respectively.

### Biochemical analysis

Fasting sera were collected from the submandibular vein using animal lancets (Medipoint, Long Island, NY) in microtainer tubes (Becton Dickinson, Franklin Lakes, NJ) from 3-month- and 8-month-old *Cx43*^+/+^ and *Cx43*^*KI/KI*^ mice. Total serum calcium and phosphate concentrations were determined using a calcium reagent kit and a phosphorus reagent set (Eagle Diagnostics, Cedar Hill, Tx). Concentrations of mouse CTX (RatLaps CTX-1 ELISA kit; Immunodiagnostic Systems, East Boldon, United Kingdom), P1NP (Rat/mouse P1NP ELISA kit; Immunodiagnostic Systems, Mountain Lakes, NJ), FGF-23 (intact), and FGF-23 (C-terminal) (Mouse/Rat FGF-23 (intact) ELISA, Mouse/Rat FGF-23 (C-Term) ELISA; Quidel, SanDiego, CA) were determined according to manufacturer instructions.

### Mouse osteoblast cultures

To study OBs, we used mouse calvarial osteoblast (mCOB) cultures and bone marrow stromal cell (BMSC) cultures. For mCOB cultures, calvariae from postnatal day 4–7 mice were digested with 0.05% trypsin (Thermo Fisher Scientific) and 0.15% collagenase (Type II; Sigma Aldrich, St. Louis, MO) at 37° C. Cells from digests 3 to 5 were collected and plated at a density of 10,000/cm^2^ in DMEM (Thermo Fisher Scientific) until confluent. Cells were maintained in osteoblast differentiation medium (α-MEM (Thermo Fisher Scientific) containing 10% fetal bovine serum (FBS; Cytiva Life Sciences, Marlborough, MA), 100 IU/mL penicillin, 100 μg/mL streptomycin (Thermo Fisher Scientific), 50 μg/mL ascorbic acid and 4mM β-glycerophosphate (Sigma Aldrich)). Medium was changed every 2–3 days. For BMSCs, bone marrow was flushed out from the shafts of femurs, tibiae and humeri of 7-to 9-week-old mice.^77^ Cells were cultured at a density of 2×10^6^ cells/well in 12-well culture plates in α-MEM containing 10% FBS, 100 IU/mL penicillin, and 100 μg/mL streptomycin. At day 3, half of the medium was changed. On day 7, cells were switched to 100% osteoblast differentiation medium containing 50 μg/mL ascorbic acid and 8 mM β-glycerophosphate (β-GP). Medium was changed every 2–3 days.

### Mouse osteoclast cultures

Bone marrow-derived macrophage (BMM) cultures were obtained from bone marrow flushed out from femora and tibiae of 7- to 9-week-old mice and cultured for 18 to 24 hours in α-MEM containing 10% FBS, 100 IU/mL penicillin, 100 μg/mL streptomycin. Non-adherent cells were collected and purified by Ficoll separation (Lymphoprep; STEMMCELL Technologies, Vancouver, Canada). Cells were cultured in α-MEM medium with 10% FBS, 100 IU/mL penicillin, 100 μg/mL streptomycin, and supplemented with M-CSF (30 ng/mL; Peprotech, Cranbury NJ) for 2 days to enrich OC progenitors followed by M-CSF and RANKL (30 ng/mL; Peprotech) treatment for 4–5 days and 9–10 days to obtain mature resting OCs (on culture plates) and actively resorbing OCs (on bone chips), respectively.

### In vitro osteoblast and osteoclast assays

For OB cultures, we determined matrix expression by ALP staining and mineral nodule formation by von Kossa staining. ALP staining was performed using a commercially available alkaline phosphatase kit (Sigma Aldrich) according to manufacturer instructions. Mineral deposition was stained with 5% silver nitrate (Sigma Aldrich).

We analyzed OC formation by TRAP staining with a commercially available kit (Sigma Aldrich).^13^ TRAP-positive stained cells ≥ 3 nuclei were counted as OCs. Actin rings of OCs were examined by rhodamine-phalloidin staining (1:40 dilution in PBS, Thermo Fisher Scientific). Nuclei were stained with Hoechst 33342 dye (trihydrochloride trihydrate; Molecular Probe, Thermo Fisher Scientific). Images were taken by a Z1 observer microscope (Carl Zeiss). For bone resorption pit assays, BMMs plated on bone chips were terminated at day 10 and bone chips were imaged using a tabletop scanning electron microscope (TM-1000, Hitachi, Tokyo, Japan). Quantitative data were obtained by Image J (National institutes of Health, NIH). Experiments were performed in triplicate.

### Quantitative real-time PCR (qPCR)

Total RNA from mCOBs, BMM cultures, and long bones (shafts of femurs and tibia without bone marrow) was isolated using TRIzol (Thermo Fisher Scientific) followed by Direct-zol RNA extraction (Zymo Research, Irvine, CA) according to manufacturer instructions. RNA was treated with DNase I and cDNA was synthesized by Superscript II reverse transcriptase (Invitrogen, Carlsbad, CA). qPCR was performed as previously described.^13^ Relative quantification of gene expression was determined by the ^ΔΔ^Ct method. Data were normalized to mouse 18S gene expression. Primer sequences are listed in Supplementary Table S1.

### Protein preparation, SDS-PAGE, and Western blotting analysis

Dissected tissues were immediately frozen by liquid nitrogen and crushed to powder. Two ends of femurs and tibiae were cut off followed by brief centrifugation to remove bone marrow. Protein was extracted by lysis buffer (1% Triton X-100, 50mM Tris (pH 7.8), 150 mM NaCl, 0.1% SDS, 10% glycerol, 0.5% deoxycholic acid) with protease and phosphatase inhibitor cocktails (Thermo Fisher Scientific). Protein concentrations were measured by BCA protein assay kit (Thermo Fisher Scientific). Equal amounts of protein were run in 10% SDS-PAGE gels. Samples were transferred to PVDF membranes (BioRad, Hercules, CA) using a wet transfer apparatus. CX43 antibody (3512) was purchased from Cell Signaling Technology, Danvers, MA. GAPDH (Santa Cruz Biotechnology, Dallas, TX) served as internal control. Bands were detected using enhanced chemiluminescent detection reagent (Azure Biosystems, Dublin, CA) and visualized by an Azure c600 imaging system (Azure Biosystems). Densitometric analyses of immunoblots were performed by Image J.

### CX43 Immunofluorescence of ovary cryosections and BMMs

Ovaries from *Cx43*^+/+^and *Cx43*^*KI/KI*^ mice were fixed in 4% paraformaldehyde (PFA, Electron Microscopy Sciences, Hatfield, PA) at 4°C for 48 hours, rinsed with 0.12M phosphate buffer, and cryo-protected with 30% sucrose at 4°C overnight. 10 μm frozen sections were cut and collected on Superfrost plus slides (Thermo Fisher Scientific). Sections were rinsed with 1x PBS, blocked with 5% normal goat serum in 1% BSA in PBS, then incubated in anti-CX43 antibody (C6219; Millipore Sigma, Burlington, MA) diluted 1:300 in blocking buffer for two hours at room temperature. After rinsing with PBS, sections were incubated in Alexa 488-goat anti-rabbit (Invitrogen) diluted 1:500 for 30 minutes at room temperature. Sections were imaged on a Zeiss Pascal confocal microscope with the same conditions for both genotypes. Multinucleated BMMs were fixed in 4% PFA for 10 minutes at room temperature. Cells were permeabilized with 0.2% Tween-20 for 3 minutes, washed with PBS, and blocked in 5% normal goat serum for 60 minutes at RT. Cells were incubated in anti-CX43 antibody (C6219; Millipore Sigma) diluted 1:500 overnight. After rinsing with PBS three times, cells were incubated with goat anti-rabbit secondary antibody (1:400) for 1 hour at RT in dark. Images were taken by a Z1 observer microscope (Carl Zeiss).

### Hemichannel and gap junction activity of Cx43

To assess the hemichannel activity, we cultured *Cx43*^+/+^ and *Cx43*^*KI/KI*^ skin fibroblasts at the density of 5000 cells per well in 96-well plates. The next day, cells were washed with PBS three times and cultured with or without 100 μM Lucifer Yellow (LY) dye in DMEM containing 3 mMM EGTA for 30 minutes. Cells were then washed and fixed in 4% PFA for 5 minutes prior to measuring dye transfer with an excitation peak at 428 nm and an emission peak at 536 nm in a microplate reader (Tecan Life Sciences, Maennedorf, Switzerland). The fluorescence reading was normalized to nuclei staining with Hoechst 33342 (excitation peak at 352 nm and emission peak at 454 nm).

To evaluate the gap junction activity, donor and recipient *Cx43*^+/+^ and *Cx43*^*KI/KI*^ skin fibroblasts were seeded at a density of 12,000/cm^2^ (day 0). The following day (day 1), recipient cells were labelled with 2.4 μM DilC18 dye (Thermo Fisher Scientific) for 2 hours. On day 2, donor cells were trypsinized and stained with 5 μM Calcein Violet AM (Thermo Fisher Scientific) for 20 minutes. Donor cells were then seeded on top of the recipient cells (Ratio of donor: recipient cells=4:1) for 2 and 5 hours. At the end points, cells were harvested and analyzed by flow cytometry using a Becton-Dickinson LSRII analyzer (Becton, Dickinson and Company, Franklin Lakes, NJ).

### Statistical Analysis

Statistical analysis was performed by one-way ANOVA followed by Bonferroni multiple comparison test, using Prism 5 (GraphPad Software, La Jolla, CA).

## Figures and Tables

**Table 1:** Dynamic and static histomorphometry of trabecular parameters in metaphyses of femurs of 13-week-old *Cx43*^*+/+*^ and *Cx43*^*KI/KI*^ male and female mice

Parameters	Male		Female	
	*Cx43^+/+^* (n=8)	*Cx43^KI/KI^* (n=8)	*Cx43^+/+^* (n=8)	*Cx43^KI/KI^* (n=8)
MAR (μm/day)	1.31±0.05	1.48±0.11	1.37±0.23	1.26±0.27
BFR (μm^3^/μm^2^/day)	0.49±0.03	0.56±0.07	0.47±0.08	0.42±0.12
AP/BS (%)	77.94±2.91	76.65±7.35^**a**^	80.9±7.12	85.86±2.29^**a**^
AP_L/BS (%)	53.83±1.12	50.64±7.30	51.42±4.16	53.16±5.18
AP_NL/BS (%)	24.73±2.04	24.96±3.25^**b**^	29.58±3.34	32.84±4.57^**b**^
TRAP/BS (%)	18.27±5.11^**a**^	13.41±2.17^**c**^	25.73±2.92^**a**^	29.74±4.85^**c**^
TRAP_L/BS (%)	11.05±2.62^**b**^	8.72±1.74^**c**^	16.96±2.52^**b**^	18.08±2.09^**c**^
TRAP_NL/BS (%)	5.48±1.47^**b**^	4.83±0.71^**c**^	10.34±1.83^**b**^	12.30±3.58^**c**^
AP_TRAP_ R/BS (%)	8.31±2.22^**a**^	6.13±1.77^**c**^	11.58±1.97^**a**^	13.13±1.55^**c**^

MAR: mineral apposition rate; BFR: bone formation rate; AP: alkaline phosphatase staining; BS: bone surface; AP/BS: AP+ surface per bone surface, L: labeling surface; AP_L/BS: AP+ over labeling surface per bone surface; NL: non-labeling surface; AP_NL /BS: AP+ over non-labeling surface per bone surface; TRAP: tartrate-resistant acid phosphatase; TRAP/BS: fraction of bone surface with TRAP label; TRAP_L/BS: proportion of mineralizing surface that is covered with the TRAP label; TRAP_NL/BS: proportion of non-mineralizing surface that is covered with the TRAP label; AP_TRAP_R/BS: proportion of bone surface where the AP, TRAP and mineralization signals are co-localized. R: alizarin complexone labeling. Data presented: mean ± SD. Significant differences between groups are noted by

a *p*<0.05,

b *p*<0.01,

c *p*<0.0001 by two-way ANOVA with Tukey’s multiple comparison test comparing *Cx43*^+/+^ to *Cx43*^*KI/KI*^ mice, male to female *Cx43*^+/+^ mice, and male to female *Cx43*^*KI/KI*^ mice.
